# Gender difference in the association of dietary intake of antioxidant vitamins with kidney function in middle-aged and elderly Japanese

**DOI:** 10.1017/jns.2020.54

**Published:** 2021-01-22

**Authors:** Akinori Hara, Hiromasa Tsujiguchi, Keita Suzuki, Fumihiko Suzuki, Tomoko Kasahara, Pham Kim Oanh, Sakae Miyagi, Takayuki Kannon, Atsushi Tajima, Takashi Wada, Hiroyuki Nakamura

**Affiliations:** 1 Department of Environmental and Preventive Medicine, Faculty of Medicine, Institute of Medical, Pharmaceutical and Health Sciences, Kanazawa University, Kanazawa, Japan; 2 Division of Nephrology, Kanazawa University Hospital, Kanazawa, Japan; 3 Department of Bioinformatics and Genomics, Faculty of Medicine, Institute of Medical, Pharmaceutical and Health Sciences, Kanazawa University, Kanazawa, Japan; 4 Department of Nephrology and Laboratory Medicine, Faculty of Medicine, Institute of Medical, Pharmaceutical and Health Sciences, Kanazawa University, Kanazawa, Japan

**Keywords:** Chronic kidney disease, Vitamin, Antioxidant, Prevention

## Abstract

Dietary intake modification is important for the treatment of chronic kidney disease (CKD); however, little is known about the association between dietary intake of antioxidant vitamins and kidney function based on gender difference. We examined the relationship of dietary intake of antioxidant vitamins with decreased kidney function according to gender in Japanese subjects. This population-based, cross-sectional study included 936 Japanese participants with the age of 40 years or older. A validated brief self-administered diet history questionnaire was used to measure dietary intakes of vitamin E and its four isoforms, vitamin A and vitamin C. Decreased kidney function was defined as estimated glomerular filtration rate <60 ml/min/1·73 m^2^. A total of 498 (53·2 %) of the study participants were women. Mean age was 62·4 ± 11·3 years. Overall, 157 subjects met the criteria of decreased kidney function. In the fully adjusted model, a high vitamin E intake is inversely associated with decreased kidney function in women (odds ratio, 0·886; 95 % confidence interval, 0·786–0·998), whereas vitamin E intake was not associated with decreased kidney function (odds ratio, 0·931; 95 % confidence interval, 0·811–1·069) in men. No significant association between dietary intake of vitamins A and C and decreased kidney function was observed in women and men. Higher dietary intake of vitamin E was inversely associated with decreased kidney function in middle-aged and older women, and the result may provide insight into the more tailored dietary approaches to prevent CKD.

## Introduction

More than 10 % of the population worldwide is affected by chronic kidney disease (CKD). It is defined by a reduced glomerular filtration rate (GFR) and increased urinary excretion of either albumin or protein, and advanced CKD requires exceptionally high costs and burden of maintenance dialysis therapy and kidney transplantation^(1)^. In addition, even the mild form of the disease is associated with increased global morbidity and mortality and is an important risk factor for cardiovascular disease (CVD)^(2)^. Recently, sex and gender-specific differences in the aetiology, mechanism and epidemiology of CKD have been recognized. It has been reported that CKD is the ninth leading cause of death (1·8 % of deaths) for women, but is not among the 10 leading causes of death for men, and the prevalence of CKD is higher in women (11·8 %) than in men (10·4 %)^(3)^. In addition, kidney function declines faster in men than in women^(3,4)^. This sex difference in kidney function decline is explained in part by protective effects of endogenous oestrogens *v.* deleterious effects of testosterone on kidney function and structure and the generally healthier lifestyles of women compared with that of men^(4)^. Although clinical evidence to support these theories is still weak, multidisciplinary approach including lifestyle modifications in consideration of sex and gender is recommended to improve this global public health issue^(3,4,5)^.

Among lifestyle interventions for people with CKD to prevent progression and CVD, dietary intake of vitamins and trace elements at conventional doses is often recommended both for persons at high risk for kidney disease and for those with established kidney disease, in addition to dietary sodium and protein reduction^(6)^. This recommendation of vitamin intake is related to the recognition that insufficient ingestion of antioxidant vitamins, such as vitamins E and C and carotenoids, in persons with CKD and that imbalance of these vitamins may contribute to a higher burden of oxidative stress, inflammation and CVD^(6)^. Recent studies have indicated that oxidative stress and chronic inflammation are also involved in the development and progression of CKD^(7)^, and levels of pro-inflammatory mediators of kidney injury and oxidative stress in the kidney are lower in female rats as compared to male rats partly due to the actions of oestrogen^(4)^. However, sex and gender differences in the association between dietary intake of antioxidant vitamins and kidney function decline in the general population have not been well studied.

Based on this background, we examined the relationship between dietary intake of the antioxidant vitamin and kidney function stratified by sex in a cohort of middle-aged and elderly people in Japan.

## Methods

### Study design and participants

This cross-sectional study was a part of the larger Shika study, a longitudinal observational community-based study that includes Shika town residents and has been described in detail elsewhere^(8,9,10)^. In this study, self-administered questionnaire was used and health examination data were collected between 2013 and 2017 in the Shika study.

**Fig. 1.**
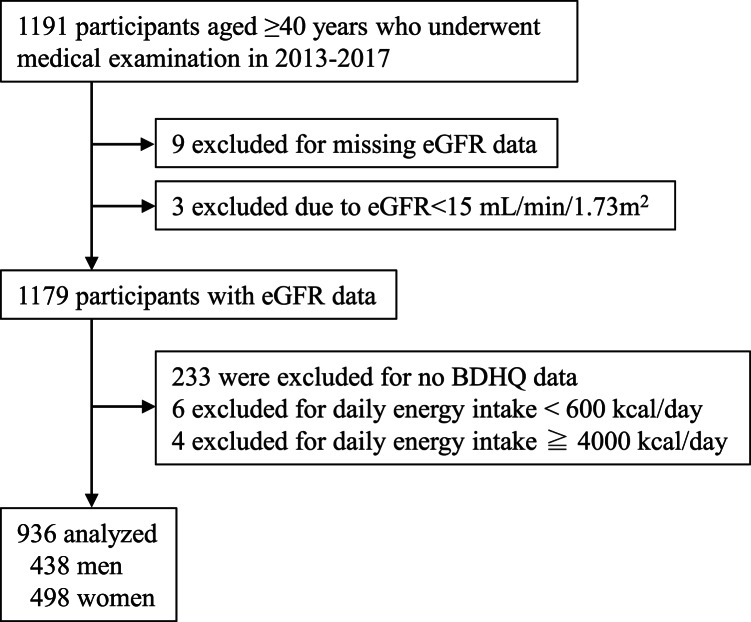
Flow diagram showing the study enrolment procedure BDHQ, brief self-administered diet history questionnaire; eGFR, estimated glomerular filtration rate.

The study enrolment procedure is outlined in Fig. 1. Nine of 1191 participants aged ≥40 years who underwent a medical examination during the study period were excluded owing to lack of data on kidney function as indicated by estimated glomerular filtration rate (eGFR) (defined later). In addition, three participants with eGFR <15 ml/min/1·73 m^2^, corresponding to end-stage kidney disease^(1,11)^, were excluded from this analysis. After excluding those with no food frequency questionnaire data (described later) and those with a daily energy intake of <600 kcal/d or >4000 kcal/d, the data of 936 individuals were finally analysed. The Shika study was approved by the Medical Ethics Committee at Kanazawa University (approval number 1491). Informed consent was obtained from all study participants.

### Assessment of kidney function

To evaluate kidney function in this Japanese population, eGFR was calculated using the following equation: eGFR (ml/min/1·73 m^2^) = 194 × serum creatinine^−1·094^ × age^−0·287^ (if female, × 0·739)^(12)^. A decreased kidney function was defined as eGFR of <60 ml/min/1·73 m^2^ according to the KDIGO 2012 Clinical Practice Guideline for the Evaluation and Management of Chronic Kidney Disease^(11)^. Serum creatinine concentrations were measured using the enzymatic method.

### Nutritional assessment

Standardized methodology was used to estimate nutritional intake from data obtained using the Japanese version of the food frequency questionnaire, which is a brief self-administered dietary history questionnaire (BDHQ)^(13)^. Information on dietary supplements was not included in the BDHQ^(13)^. The validity of BDHQ has been confirmed by other studies, which is considered to have a satisfactory ranking ability for many nutrients among the Japanese population^(14,15)^. Daily caloric intake and intake of protein, lipids, carbohydrates and antioxidant vitamins E, A and C were estimated from the results of the BDHQ. Among the vitamin E family, α-tocopherols are known as a predominant form of vitamin E found in blood and tissues and previous studies were performed using mainly α-tocopherol, recent studies have revealed that other forms of vitamin E, such as β-, γ- and δ-tocopherols, have other distinctive actions besides antioxidants^(16,17)^. Based on these findings, we included information on dietary intake of β-, γ- and δ-tocopherols, as well as α-tocopherol in this study. Vitamin A intake was expressed as retinol equivalent. To obtain improved values which correlated better with the dietary record than crude values, nutritional intake was adjusted for energy using the density method as a percentage of the daily intake of energy-containing nutrients^(8,13)^.

### Other variables

Blood pressure (BP) was measured at rest in a sitting position. Hypertension was defined as systolic BP of ≥140 mm Hg, diastolic BP of ≥90 mm Hg or being on antihypertensive medication. Weight, height and glycated haemoglobin (HbA_1c_) were measured during follow-up appointments. Body mass index (BMI) was expressed as kg/m^2^. Diabetes was defined as being on antidiabetic medication or HbA_1c_ of ≥6·5 %, which is the cutoff value used by the Japan Diabetes Society to diagnose diabetes mellitus^(18)^. Dyslipidemia was defined as being on lipid-lowering agents or total cholesterol levels of ≥240 mg/dl^(19,20)^. Urine dipstick test was performed using spot urine specimens, and proteinuria was recorded as negative, 1+, 2+, 3+ and 4+. Proteinuria was defined as dipstick proteinuria ≥1+ (corresponded to ≥300 mg/g).

Other variables, such as age, sex, smoking status, frequency of exercise and alcohol consumption, were assessed using self-administered questionnaires. Smoking status was classified as nonsmoker, ex-smoker or current smoker^(9)^. Habitual alcohol consumption was defined as drinking more than 1 glass of sake (22 g ethanol) per day at least 3 times a week^(9)^. Frequency of exercise was estimated as follows. Participants were asked whether or not they had exercise of more than 30 min at least twice a week during the previous year or performed tasks, such as walking, cleaning and carrying baggage for more than 1 h per d. Participants who responded in the affirmative to either of these questions were considered as having exercise at an adequate level^(9)^.

### Statistical analysis

Descriptive characteristics at baseline were compared according to sex and/or the presence or absence of eGFR of <60 ml/min/1·73 m^2^. Comparison of the means of continuous variables was examined using Student's *t*-test, and comparison of proportions of categorical variables was examined using *χ*^2^ test. A two-way analysis of variance (ANOVA) was used to examine the interaction in each intake of antioxidant vitamin between sex and the presence or absence of eGFR of <60 ml/min/1·73 m^2^. The relationship between eGFR of <60 ml/min/1·73 m^2^ and each intake of antioxidant vitamin was examined using multivariable logistic regression analysis according to gender. There are two models with different adjustments for the following potential confounders based on previous studies^(19,21)^: model 1 adjusted for age, BMI, diabetes and hypertension and model 2 adjusted for all variables in model 1, plus smoking status, dyslipidemia and fat intake. As for the association with eGFR of <60 ml/min/1·73 m^2^, the relationship between proteinuria and each intake of antioxidant vitamin was examined using a multivariable logistic regression analysis according to gender based on the previous report^(22)^.

SPSS version 23 (IBM Corp., Tokyo, Japan) was used for all analyses. *P*<0·05 was considered statistically significant.

## Results

### Participant characteristics according to kidney function

Participant characteristics and nutrients intake are summarized according to sex and kidney function in Table 1. Among 936 participants (62·4 (SD, 11·3) years old), 47·8 % (*n* 438) were men and 157 (16·8 %) had CKD. Age, ratio of hypertensive subjects, BMI and SBP were higher in the decreased kidney function group than in the nondecreased kidney function group (Supplementary Table S1 of Supplementary material). Ratio of smoking habits was significantly higher in the nondecreased kidney function group than in the decreased kidney function group, whereas consumption of carbohydrate was lower in the nondecreased kidney function group than in the decreased kidney function group. There was no difference in the frequency of exercise, drinking habits and the consumption of tocopherols and vitamins A and C between the groups.

**Table 1.** Participant characteristics in different kidney function groups according to gender

	Men			Women		
	Nondecreased kidney function (*n* 358)	Decreased kidney function (*n* 80)	*P* value	Nondecreased kidney function (*n* 421)	Decreased kidney function (*n* 77)	*P* value
Age (years)	60·6 ± 10·8	68·9 ± 10·2	<0·001	61·3 ± 11·0	70·2 ± 10·9	<0·001
Current smoking (*n*)	134 (37·4 %)	12 (15·0 %)	<0·001	33 (7·8 %)	2 (2·6 %)	0·098
Exercise habit (*n*)	76 (21·2 %)	20 (25·0 %)	0·46	67 (15·9 %)	18 (23·4 %)	0·11
Drinking habit (*n*)	210 (58·7 %)	38 (47·5 %)	0·069	40 (9·5 %)	7 (9·1 %)	0·91
BMI (kg/m^2^)	23·7 ± 3·0	25·0 ± 3·4	0·001	22·7 ± 3·3	22·7 ± 3·3	0·95
SBP (mmHg)	140·6 ± 19·2	147·1 ± 20·1	0·007	134·7 ± 19·0	142·6 ± 20·6	0·001
DBP (mmHg)	82·3 ± 11·9	82·5 ± 12·3	0·90	77·9 ± 10·7	76·9 ± 12·3	0·44
Hypertension (*n*)	225 (62·8 %)	64 (80·0 %)	0·003	206 (48·9 %)	49 (63·6 %)	0·018
Haemoglobin (g/dl)	15·2 ± 1·2	15·3 ± 1·5	0·62	13·3 ± 1·3	13·3 ± 1·3	0·80
Total cholesterol (mg/dl)	207·6 ± 32·7	208·6 ± 31·9	0·80	218·1 ± 34·7	215·6 ± 26·5	0·47
Dyslipidemia (*n*)	93 (26·0 %)	23 (28·8 %)	0·61	155 (36·8 %)	23 (29·9 %)	0·24
HbA1c (%)	5·9 ± 0·8	6·0 ± 0·6	0·53	5·9 ± 0·6	5·9 ± 0·5	0·81
Diabetes (*n*)	59 (16·5 %)	18 (22·5 %)	0·20	41 (9·7 %)	6 (7·8 %)	0·59
Serum creatinine (mg/dl)	0·82 ± 0·10	1·13 ± 0·20	<0·001	0·61 ± 0·08	0·83 ± 0·12	<0·001
eGFRcr (mL/min/1.73m^2^)	75·8 ± 10·9	51·8 ± 7·3	<0·001	77·2 ± 12·0	53·4 ± 6·9	<0·001
Proteinuria (*n*)^a^	17 (4·8 %)	11 (13·9 %)	0·003	11 (2·6 %)	9 (12·0 %)	<0·001
Dietary intake						
Daily energy intake (kcal)	2116 ± 610	1947 ± 564	0·023	1657 ± 530	1709 ± 498	0·43
Protein (%energy)	14·3 ± 3·0	14·9 ± 3·4	0·12	16·0 ± 3·1	15·5 ± 3·6	0·23
Lipids (%energy)	22·3 ± 5·6	24·1 ± 6·4	0·015	26·4 ± 5·6	24·7 ± 6·0	0·015
Carbohydrate (%energy)	53·3 ± 8·8	53·3 ± 9·3	0·96	54·7 ± 8·1	57·9 ± 8·4	0·001
Vitamin A (μg/1000kcal)	342·1 ± 396·3	345·6 ± 178·3	0·94	396·3 ± 404·0	368·2 ± 183·0	0·55
Total tocopherols (mg/1000 kcal)	11·2 ± 3·14	11·88 ± 3·2	0·086	13·41 ± 3·16	12·31 ± 3·34	0·006
α-tocopherol (mg/1000 kcal)	3·38 ± 0·98	3·61 ± 0·96	0·063	4·22 ± 1·05	4·03 ± 1·25	0·17
β-tocopherol (mg/1000 kcal)	0·18 ± 0·05	0·19 ± 0·05	0·26	0·20 ± 0·05	0·18 ± 0·04	<0·001
γ-tocopherol (mg/1000 kcal)	6·07 ± 1·94	6·40 ± 2·02	0·18	7·13 ± 1·99	6·39 ± 1·92	0·003
δ-tocopherol (mg/1000 kcal)	1·57 ± 0·48	1·69 ± 0·53	0·064	1·86 ± 0·49	1·70 ± 0·49	0·010
Vitamin C (mg/1000 kcal)	48·84 ± 25·76	55·30 ± 28·86	0·048	70·19 ± 31·41	74·24 ± 35·36	0·31

BMI, body mass index; Cr, creatinine; DBP, diastolic blood pressure; eGFR, estimated glomerular filtration rate; HbA_1c_, glycated haemoglobin; SBP, systolic blood pressure.

Data for continuous variables are expressed as the means and standard deviations.

a *n* 927

### Participant characteristics according to sex and kidney function

In male subjects, the ratios of hypertensive subjects, age, BMI and fat intake were higher in the decreased kidney function group than in the nondecreased kidney function group (Table 1). No significant difference in vitamin intake except for vitamin C was found between the groups.

In female subjects, the ratios of hypertensive subjects, age and carbohydrate intake were higher in the decreased kidney function group than in the nondecreased kidney function group, and fat intake was lower in the decreased kidney function group than in the nondecreased kidney function group (Table 1). In contrast to male subjects, there was a significant difference in tocopherol intake between the groups; the decreased kidney function group consumed fewer tocopherols excluding α form than the nondecreased kidney function group. Statistically, there was a significant interaction between the kidney function groups and sex on the intake of tocopherols (*P* = 0·024 for α-tocopherol, *P* = 0·001 for β-tocopherol, and *P* = 0·002 for γ- and δ-tocopherols). This result suggests that there is a relationship between the intake of tocopherols and decreased kidney function according to sex. For vitamins A and C, no significant interactions were observed (data not shown).

### Relationship between antioxidant vitamins intake and kidney function according to sex

The relationship between the consumption of antioxidant vitamins and kidney function is presented in Table 2. Following the results presented in Table 1, which indicated the interaction between the kidney function groups and sex, subjects were stratified according to sex. High consumption of tocopherols was inversely correlated with decreased kidney function in female subjects after adjustment for confounding factors, although α- and γ-tocopherol intakes were not statistically significant. Intake of vitamins A and C was not associated with decreased kidney function. On the other hand, there were no significant associations between the intake of tocopherols or vitamins A or C and decreased kidney function in male subjects.

**Table 2.** Association between dietary intake of antioxidant vitamins and decreased kidney function by gender

Gender	Vitamins	Model 1			Model 2		
		OR	95 % CI	*P* value	OR	95 % CI	*P* value
Men	Total T	1·069	0·985–1·160	0·11	0·931	0·811–1·069	0·31
	αT	1·090	0·835–1·423	0·53	0·577	0·371–0·897	0·015
	βT	159·404	0·835–30427·707	0·058	1·748	0·002–1825·288	0·88
	γT	1·138	0·997–1·298	0·055	0·987	0·809–1·205	0·90
	δT	1·331	0·803–2·207	0·27	0·842	0·448–1·579	0·59
	Vitamin A	0·999	0·998–1·001	0·34	0·998	0·997–1·000	0·070
	Vitamin C	0·997	0·987–1·007	0·50	0·989	0·979–1·000	0·059
Women	Total T	0·912	0·840–0·990	0·027	0·886	0·786–0·998	0·047
	αT	0·820	0·649–1·035	0·095	0·824	0·595–1·142	0·25
	βT	0·001	0·000–0·304	0·017	0·001	0·000–0·855	0·045
	γT	0·868	0·758–0·994	0·041	0·853	0·711–1·023	0·087
	δT	0·519	0·300–0·898	0·019	0·519	0·276–0·975	0·041
	Vitamin A	0·999	0·998–1·001	0·30	0·999	0·998–1·001	0·47
	Vitamin C	0·998	0·990–1·006	0·62	0·999	0·991–1·007	0·77

CI, confidence interval; T, tocopherol; OR, odds ratio.

Model 1: adjusted for age, BMI, diabetes, hypertension.

Model 2: all variables in model 1, plus smoking, dyslipidemia, and fat intake.

### Relationship between antioxidant vitamins intake and proteinuria according to sex

Multivariable analysis to further examine the association between dietary intake of antioxidant vitamin and proteinuria was presented in Supplementary Table S2 of Supplementary material. There was no significant relationship between intake of antioxidant vitamin and proteinuria in both genders except for the negative association of vitamin C intake and proteinuria in women.

## Discussion

In this study, we found that lower intake of tocopherols was associated with lower kidney function independent of other important confounders in community-dwelling middle-aged and older Japanese women, whereas this association was not observed in the middle-aged and older men. In addition, the association between retinol and vitamin C intake and kidney function was not observed in both men and women.

The present results for vitamin E are consistent with previous studies indicating a protective association between dietary vitamin E intake or its serum levels and kidney function decline. A previous epidemiological study in the US Third National Health and Nutrition Examination Survey reported that serum vitamin E levels were associated with decreased kidney function in the non-Hispanic white population^(19)^. Another study in Iran reported that dietary intake of vitamin E was associated with a decreased risk of incident CKD^(21)^. In human interventional studies, vitamin E supplementation decreased the risk of contrast-induced acute kidney injury in CKD patients and provided a positive effect on kidney function in diabetic patients^(16,23)^. On the other hand, there are some conflicting results on the association between vitamin E intake and kidney function^(23)^. In addition, a recent epidemiological study reported that there was no association between serum vitamin E concentration and eGFR decline in young US adults^(24)^. Interpretation of these results may need to be considered to the heterogeneity of the enrolled population, such as differences in sex as well as age, race/ethnicity and comorbidities.

Among four forms of vitamin E, lower intake of β-, γ- and δ-tocopherols, but not α-tocopherol, was significantly associated with lower kidney function in women. Vitamin E refers to a group of fat-soluble compounds with antioxidant capacity, including α-, β-, γ- and δ-tocopherols and α-, β-, γ- and δ-tocotrienols showing different biological properties^(16,17)^. Although overall intake of vitamins E and C decreased kidney damage by reducing kidney superoxide release, arterial pressure and tissue inflammation concomitant with improving GFR and effective plasma flow in an animal model of hypertension-induced kidney injury^(25)^, our present results may provide us further insights into the mechanisms of the beneficial effects of β-, γ- and δ-tocopherols on kidney function and metabolic risk factors for CKD. In subjects with metabolic syndrome, TNF-α levels in blood decreased by α-tocopherol supplementation alone and in combination with γ-tocopherol, whereas nitrotyrosine levels in urine, a marker of reactive nitrogen species, were decreased by γ-tocopherol supplementation alone or in combination with α-tocopherol, suggesting more potent ability of decreasing nitrosative stress in γ-tocopherol than in α-tocopherol^(26)^. γ-tocopherol also acts as a trap for other electrophilic mutagens, inhibits smooth muscle cell proliferation and decreases platelet aggregation and delays thrombus formation in experimental studies^(17)^. Furthermore, anti-inflammatory effects of γ-tocopherol and δ-tocopherols may be mediated by its effect on inhibiting cyclooxygenase 2- and 5-lipoxigenase-mediated eicosanoids and suppressing NF-kB and JAK-STAT6 or JAK-STAT3 signalling pathways in various cell types including macrophages^(16,17)^. Moreover, a recent *in vitro* study revealed that δ-tocopherol increased the gene expression of peroxisome proliferator-activated receptor γ co-activator-1α and uncoupling protein 1 gene in adipocytes, suggesting the potential ability of this form to enhance energy consumption in adipocytes^(27)^. Collectively, although further studies are needed to confirm the mechanism of action, these anti-inflammatory and favourable metabolic effects and antioxidant capacity of each form of vitamin E and its metabolites may synergistically serve to protect kidney function.

The association of dietary intake of vitamin E with kidney function was observed only in women. Gender difference in dietary intake of and plasma and/or tissue concentrations of vitamin E has been recognized by animals and human studies, but the underlying mechanism remains to be examined^(28,29,30,31)^. Overall, a recent review indicates that possible mechanisms for sex differences in CKD progression include direct effects of sex steroid on the kidney, sex differences in NO metabolism and oxidative stress and gender-differential effect of comorbidities and lifestyle risk factors^(4)^. With regard to vitamin E, a recent study revealed that urinary levels of α-carboxymethylhydroxychroman, a final metabolite of α-tocopherol, were higher in female than in male rats and decreased by testosterone administration, although underlying mechanism was not indicated^(29)^. Furthermore, in the kidney of mice, substantial higher concentrations in most of the inflammation biomarkers, such as MCP-1, IL-6 and TNF-α were observed for the high dose of vitamin E, but only in male mice^(32)^. This evidence suggests that there may be gender difference in both vitamin E metabolism and its effect on the development and progression of kidney diseases. Further studies are needed to examine whether a new evaluation of the recommended daily intake of vitamin E by gender has to be considered.

This study has several limitations. First, the design was cross-sectional, implying that a causal relationship could not be established between intake of antioxidant vitamin and CKD. Therefore, whether or not dietary intake of antioxidant vitamins can prevent the development of kidney function decline in the general population has yet to be determined. Second, in addition to the lack of inclusion of objective markers of vitamin intake, the data on the daily intake of energy, protein, lipids and vitamins were obtained from BDHQ, which contains a limited number of food and beverage items and does not provide an accurate estimate of absolute dietary intake. Furthermore, these data may have been affected by recall bias. Finally, there is a possibility of both selection bias and treatment effect bias, given that the Shika study participants are volunteers and might be more health conscious than the general population.

In conclusion, this cross-sectional study has reported that dietary vitamin E intake inversely associated with lower kidney function in Japanese women aged ≥40 years. Further basic and longitudinal studies using objective markers of vitamin intake are needed to confirm this relationship and to investigate whether specific dietary intervention can prevent the development of CKD.

## Ethics approval

All procedures performed in studies involving human participants were in accordance with the ethical standards of the medical ethics committee of Kanazawa University at which the studies were conducted (approval number 1491) and with the 1964 Helsinki declaration and its later amendments or comparable ethical standards.

## Supplementary material

For supplementary material accompanying this paper visit https://doi.org/10.1017/jns.2020.54. click here to view supplementary material
