# Supplementary material for: Gender difference in the association of dietary intake of antioxidant vitamins with kidney function in middle-aged and elderly Japanese
Source: J Nutr Sci. 2021 Jan 22;10:e2. doi: 10.1017/jns.2020.54 (PMC8057365; doi:10.1017/jns.2020.54)
Supplement: Supplementary file 1 [file S2048679020000543sup001.docx]

**Supplementary Table 1. Participant characteristics in different kidney function groups**

|  | Nondecreased  kidney function  (n = 779) | Decreased  kidney function  (n = 157) | *P* value |
| --- | --- | --- | --- |
| Age (years) | 61.0 ± 10.9 | 69.5 ± 10.5 | <0.001 |
| Men (n) | 358 (46.0%) | 80 (51.0%) | 0.25 |
| Current smoking (n) | 167 (21.4%) | 14 (8.9%) | <0.001 |
| Exercise habit (n) | 143 (18.4%) | 38 (24.2%) | 0.091 |
| Drinking habit (n) | 250 (32.1%) | 45 (28.7%) | 0.40 |
| BMI (kg/m^2^) | 23.2 ± 3.2 | 23.9 ± 3.5 | 0.018 |
| SBP (mmHg) | 137.5 ± 19.3 | 144.9 ± 20.4 | <0.001 |
| DBP (mmHg) | 79.9 ± 11.5 | 79.7 ± 12.6 | 0.84 |
| Hypertension (n) | 431 (55.3%) | 113 (72.0%) | <0.001 |
| Hemoglobin (g/dL) | 14.2 ± 1.5 | 14.3 ± 1.7 | 0.39 |
| Total cholesterol (mg/dL) | 213.3 ± 34.2 | 212 ± 29.5 | 0.68 |
| Dyslipidemia (n) | 248 (31.8%) | 46 (29.3%) | 0.53 |
| HbA1c (%) | 5.9 ± 0.7 | 5.9 ± 0.6 | 0.47 |
| Diabetes (n) | 100 (12.8%) | 24 (15.3%) | 0.41 |
| Serum creatinine (mg/dL) | 0.71 ± 0.14 | 0.98 ± 0.23 | <0.001 |
| eGFRcr (mL/min/1.73m^2^) | 76.6 ± 11.5 | 52.6 ± 7.2 | <0.001 |
| Proteinuria (n)* | 28 (3.6%) | 20 (13.0%) | <0.001 |
| Daily energy intake (kcal) | 1868 ± 612 | 1830 ± 544 | 0.47 |
| Protein (% energy) | 15.2 ± 3.2 | 15.2 ± 3.5 | 0.95 |
| Lipids (% energy) | 24.5 ± 6.0 | 24.4 ± 6.2 | 0.76 |
| Carbohydrate (% energy) | 54.1 ± 8.4 | 55.6 ± 9.1 | 0.048 |
| Vitamin A (μg/1000kcal) | 371.4 ± 401.14 | 356.68 ± 180.40 | 0.65 |
| Total tocopherols (mg/1000 kcal) | 12.39 ± 3.34 | 12.09 ± 3.27 | 0.29 |
| α-tocopherol (mg/1000 kcal) | 3.83 ± 1.10 | 3.82 ± 1.13 | 0.86 |
| β-tocopherol (mg/1000 kcal) | 0.19 ± 0.05 | 0.18 ± 0.05 | 0.064 |
| γ-tocopherol (mg/1000 kcal) | 6.64 ± 2.03 | 6.39 ± 1.97 | 0.16 |
| δ-tocopherol (mg/1000 kcal) | 1.73 ± 0.51 | 1.69 ± 0.51 | 0.45 |
| Vitamin C (mg/1000 kcal) | 60.38 ± 30.83 | 64.59 ± 33.48 | 0.12 |

Data for continuous variables are expressed as the mean±standard deviation.

*n = 927

*BMI*, body mass index; *Cr*, creatinine; *DBP*, diastolic blood pressure; *eGFR*, estimated glomerular filtration rate; *HbA_1c_*, glycated hemoglobin; *SBP*, systolic blood pressure

**Supplementary Table 2.** Association between antioxidant vitamins intake and proteinuria by gender

| Gender | Vitamins | Odds ratio | 95% confidence interval | *P* value |
| --- | --- | --- | --- | --- |
| Men | Total T | 0.984 | 0.868–1.115 | 0.80 |
|  | αT | 1.054 | 0.700–1.586 | 0.80 |
|  | βT | 0.803 | 0.000–2103.246 | 0.96 |
|  | γT | 0.969 | 0.791–1.186 | 0.76 |
|  | δT | 0.708 | 0.315–1.589 | 0.40 |
|  | Vitamin A | 1.000 | 0.999–1.001 | 0.78 |
|  | Vitamin C | 0.990 | 0.974–1.006 | 0.23 |
|  |  |  |  |  |
| Women | Total T | 0.958 | 0.832–1.103 | 0.55 |
|  | αT | 0.753 | 0.483–1.172 | 0.21 |
|  | βT | 0.238 | 0.000–2327.877 | 0.76 |
|  | γT | 0.983 | 0.787–1.227 | 0.88 |
|  | δT | 0.777 | 0.313–1.930 | 0.59 |
|  | Vitamin A | 0.998 | 0.995–1.001 | 0.19 |
|  | Vitamin C | 0.978 | 0.960–0.997 | 0.022 |

*T*, tocopherol.

Odds ratio was adjusted for age, BMI, diabetes, and hypertension
